# Perioperative mortality after hemiarthroplasty related to fixation method: A study based on the Australian Orthopaedic Association National Joint Replacement Registry

**DOI:** 10.3109/17453674.2011.584208

**Published:** 2011-07-08

**Authors:** Darren J Costain, Sarah L Whitehouse, Nicole L Pratt, Stephen E Graves, Philip Ryan, Ross W Crawford

**Affiliations:** ^1^Institute of Health and Biomedical Innovation, Queensland University of Technology, Brisbane; ^2^Orthopaedic Research Unit, Prince Charles Hospital, Queensland; ^3^Data Management and Analysis Centre, Discipline of Public Health, University of Adelaide; ^4^Australian Orthopaedic Association National Joint Replacement Registry, Adelaide, Australia

## Abstract

**Background and purpose:**

The appropriate fixation method for hemiarthroplasty of the hip as it relates to implant survivorship and patient mortality is a matter of ongoing debate. We examined the influence of fixation method on revision rate and mortality.

**Methods:**

We analyzed approximately 25,000 hemiarthroplasty cases from the AOA National Joint Replacement Registry. Deaths at 1 day, 1 week, 1 month, and 1 year were compared for all patients and among subgroups based on implant type.

**Results:**

Patients treated with cemented monoblock hemiarthroplasty had a 1.7-times higher day-1 mortality compared to uncemented monoblock components (p < 0.001). This finding was reversed by 1 week, 1 month, and 1 year after surgery (p < 0.001). Modular hemiarthroplasties did not reveal a difference in mortality between fixation methods at any time point.

**Interpretation:**

This study shows lower (or similar) overall mortality with cemented hemiarthroplasty of the hip.

The frequency of hip fractures is increasing with our ageing population, with an annual incidence of between 1.4 and 5 per 103 per year (Lonnroos et al. 2006, Icks et al. 2008, Varez-Nebreda et al. 2008). Health model projections have estimated that 6.3 million hip fractures will occur annually worldwide within the next 40 years (Cooper et al. 1992), imposing a significant economic health burden. There is a large reported perioperative mortality rate in this population, ranging from 2.4% to 8.2% at 1 month (Parvizi et al. 2001, Radcliff et al. 2008) and over 25% at 1 year (Elliott et al. 2003, Jiang et al. 2005). Furthermore, it was recently reported that the current mortality rate is higher now than 25 years ago (Vestergaard et al. 2007a). Today, it is generally accepted that displaced intracapsular fractures are best treated with arthroplasty rather than internal fixation (Keating et al. 2006, Leighton et al. 2007). In the at-risk population, however, multiple comorbidities are common and the best form of component fixation is in question.

Bone cement implantation syndrome is a well-described complication of cemented hip arthroplasty. It is characterized by a systemic drop in systolic blood pressure, hypoxemia, pulmonary hypertension, cardiac dysrhythmias, and occasionally cardiac arrest and death (Rinecker 1980, Orsini et al. 1987, Parvizi et al. 1999). The prevailing theory to explain the pathophysiology of this phenomenon is embolism of fat, marrow contents, bone, and to some degree methylmethacrylate to the lung (Rinecker 1980, Elmaraghy et al. 1998, Parvizi et al. 1999, Koessler et al. 2001). An increased degree of pulmonary insult with fat microemboli has been demonstrated (mostly in randomized controlled trials) during insertion of a cemented femoral stem rather than an uncemented implant (Orsini et al. 1987, Ries et al. 1993, Christie et al. 1994, Pitto et al. 1999), presumably due to increased intramedullary femoral canal pressures in the cemented group (Kallos et al. 1974, Orsini et al. 1987). These pressures can be reduced by the use of distal venting holes in the femur during stem insertion (Engesæter et al. 1984). It has been shown previously by single-institutional review that patients undergoing cemented hip arthroplasty have a higher intraoperative mortality rate relative to uncemented arthroplasty, presumably due to a reduced incidence of fat embolism in the latter group (Parvizi et al. 1999). The increased mortality risk was also present at 30 days in the treatment of acute fractures with cemented arthroplasty, also from a single-institutional review (Parvizi et al. 2004). Although cement-related mortality is rare (Dearborn and Harris 1998, Parvizi et al. 1999, 2001, 2004, Weinrauch et al. 2006), it is a devastating complication—often reported through observational studies or literature reviews. Proponents of uncemented hip arthroplasty often cite this concern to support their reluctance to use cemented hip arthroplasty in both elective procedures and fracture management. However, many different types of studies have been unable to identify any increased mortality risk with the use of cement (Lausten and Vedel 1982 (observational), Emery et al. 1991 (RCT), Lo et al. 1994 (observational), Khan et al. 2002a,b (literature review), Parker and Gurusamy 2004 (literature review)) and others have shown a decrease in mortality at 30 days when cement is used (Foster et al. 2005).

Cemented hip hemiarthroplasty appears to offer improved rate of return to baseline function, reduced postoperative pain, and superior long-term survivorship relative to uncemented arthroplasty (Khan et al. 2002a, b, Parker and Gurusamy 2004). We reasoned that failure to return to baseline function after hemiarthroplasty may be another risk factor for perioperative mortality (Hannan et al. 2001, Braithwaite et al. 2003). Lower revision rates for cemented prostheses and increased mortality at revision surgery contribute further to reducing the overall mortality risk. We evaluated the relationship between the method of fixation of hip arthroplasty and perioperative mortality using a large national joint replacement registry.

## Patients and methods

Data pertaining to patient age, implant type, fixation method, and patient location were obtained from the Australian Orthopaedic Association (AOA) National Joint Replacement Registry (NJRR). Mortality information was obtained by patient matching with the National Death Index (NDI) from the Australian Institute of Health and Welfare. The outcome of interest was mortality at 1 day, 1 week, 1 month, and 1 year after surgery. Data were then stratified by implant type to examine the effect of cement fixation within monoblock and modular implant procedures.

The AOA NJRR identified patient selection differences for implant type based on demographic data. As patient comorbidities are not captured in the AOA NJRR, these demographics were used as a surrogate measure for different patient populations in an effort to adjust for bias in the comparison of fixation method. We hypothesized that monoblock components are usually reserved for more elderly, lower-demand patients with more comorbidities and that modular prosthesis implants are used in healthier patients with expected longer survival.

Data in the AOA NJRR are collected at the time of surgery using a standard paper-based form, with methods described in more detail elsewhere (Conroy et al. 2008, AOA 2009). Each hospital subsequently forwards these forms to the registry for data entry. Forms with incomplete or inconsistent data are followed up by the registry with the hospital concerned. Cases where forms have not been completed are identified by verification of registry data using government hospitalization separation data.

### Statistics

Mortality rates were compared between cemented and uncemented prostheses using a time-dependent Cox proportional-hazards model. For each model, the assumption of proportional hazards was checked analytically by inspecting the graph of log(log(survival)) plotted against log of survival time. Time points were selected a priori based on clinical importance, and hazard ratios were then calculated for each selected time period. All analyses were adjusted for age and sex as measured at the date of the primary procedure. All analyses were performed using SAS software version 9.1.

### Ethics

Local ethical approval was not required from our institution, as this study was purely data-driven and used de-identified national data. A formal request was made to the Australian Orthopaedic Association (AOA) National Joint Replacement Registry (NJRR) for access to the national de-identified data.

## Results

### Patient demographics

12,804 patients were treated with uncemented hemiarthroplasty and 12,935 were treated with cemented hemiarthroplasty. No statistically significant differences in demographic characteristics between the methods of fixation were detected among the different groups (Table 1).

**Table 1.** Patient demographics for hemiarthroplasty procedures

Type of component	Total		Age		% Females
		< 70	71–80	> 80	
Monoblock					
Cemented	3,634	169 (4.7%)	946 (26.0%)	2,519 (69.3%)	74
Uncemented	10,362	420 (4.1%)	2,550 (24.6%)	7,392 (71.3%)	74
Subtotal	13,996	589	3,496	9,911	
Modular					
Cemented	9,301	1,518 (16.3%)	3,233 (34.8%)	4,550 (49.6%)	74
Uncemented	2,442	446 (18.3%)	750 (30.7%)	1,246 (51.0%)	72
Subtotal	11,743	1,964	3,983	5,796	
Total	25,739	2,553	7,479	15,707	

### Perioperative mortality

**Figure 1.**
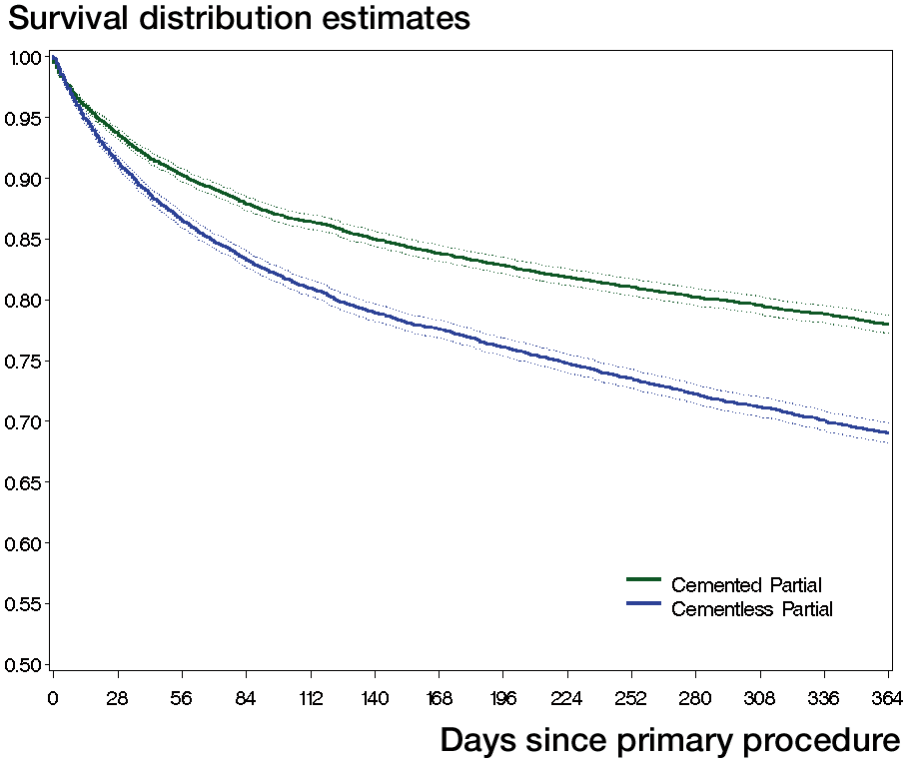
All-cause mortality in cemented and uncemented hemiarthroplasty patients.

Kaplan Meier survival estimates by postoperative days are shown in Figure 1 and hazard ratios are detailed in Table 2. There was an increased risk of perioperative mortality in patients treated with uncemented hemiarthroplasty at 1 week (p = 0.02), 1 month (p = 0.03), and 1 year (p < 0.001) postoperatively. Conversely, there was a greater risk of perioperative mortality in the first postoperative day in patients treated with cemented components (p < 0.001), suggesting that at-risk patients are more likely to succumb early if cement is used. However, most patients receiving cemented components were treated with modular components (9,301 of 12,935; 72%), whereas most patients receiving uncemented components received a monoblock prosthesis (10,362 of 12,804; 81%). We were therefore interested in further characterizing the role of fixation method in different patient groups, to identify the true effect of cement on mortality.

**Table 2.** Hazard ratios (HRs) for risk of death, according to kind of fixation, for all hemiarthroplasties

	HR (95% CI) (cementless vs. cemented)	p-value	No. of deaths	
			Cemented	Cementless
			(n = 12,935)	(n = 12,804)
1 day	0.59 (0.43–0.79)	0.0005	109	70
1 week	1.36 (1.05–1.74)	0.02	345	384
1 month	1.27 (1.03–1.58)	0.03	860	1,170
1 year	1.37 (1.29–1.49)	< 0.001	2,680	3,794

### Cemented vs. uncemented monoblock components

**Figure 2.**
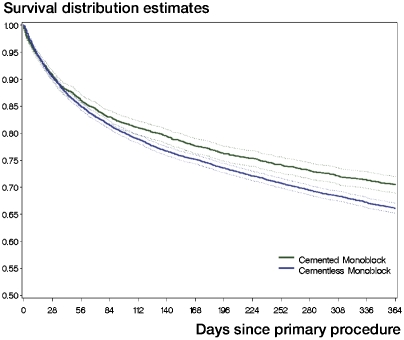
All-cause mortality for cemented and uncemented monoblock hemiarthroplasty.

10,362 patients were treated with uncemented monoblock implants and 3,634 patients received cemented monoblock implants. The mortality rate was higher at day 1 when cemented monoblock implants were used (p < 0.001). This has been further detailed—per day for the first postoperative week—in Table 3. However, this difference between groups was no longer statistically significant at 1 week or 1 month. By 1 year, the death rate had reversed with a favorable survival for patients treated with cemented implants (p < 0.001) (Figure 2 and Table 4).

**Table 3.** Hazard ratios (HRs) for day of operation to day 6 for risk of death, according to kind of fixation, for monoblock hemiarthroplasty

	Cemented			Cementless			
	No. at risk at start of period	Deaths	Cumulative survival (95% CI)	No. at risk at start of period	Deaths	Cumulative survival (95% CI)	HR (95% CI) (cementless vs. cemented)
0	3,634	0	100	10,362	0	100	
1	3,582	46	99.3 (99.0–99.6)	10,299	62	99.9 (99.8–99.9)	0.47 (0.32–0.68)
2	3,557	70	98.7 (98.3–99.0)	10,258	102	99.4 (99.2–99.5)	0.57 (0.35–0.95)
3	3,540	85	98.1 (97.6–98.5)	10,196	161	99.0 (98.8–99.2)	1.35 (0.76–2.37)
4	3,524	100	97.7 (97.1–98.1)	10,143	212	98.4 (98.2–98.7)	1.17 (0.66–2.07)
5	3,508	113	97.2 (96.7–97.7)	10,092	259	98.0 (97.7–98.2)	1.24 (0.67–2.29)
6	3,497	123	96.9 (96.3–97.4)	10,055	295	97.5 (97.2–97.8)	1.24 (0.61–2.49)

**Table 4.** Hazard ratios (HRs) for risk of death, according to kind of fixation, for monoblock hemiarthroplasty

	HR (95% CI) (cementless vs. cemented)	p-value	No. of deaths	
			Cemented	Cementless
			(n = 3,634)	(n = 10,362)
1 day	0.47 (0.32–0.68)	< 0.001	46	62
1 week	1.16 (0.81–1.66)	0.4	138	340
1 month	0.95 (0.71–1.26)	0.7	359	1,051
1 year	1.23 (1.13–1.34)	< 0.001	1,015	3,413

**Figure 3.**
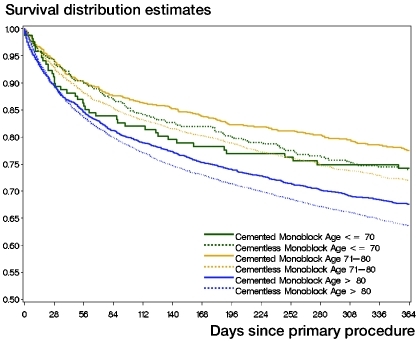
All-cause mortality in cemented and uncemented monoblock hemiarthroplasty patients stratified by age.

As comorbidities increase with age, we hypothesized that if cement was a risk factor for perioperative mortality, the relationship would be more evident in the elderly patients treated with cemented hemiarthroplasty. To investigate this relationship, we analyzed this cohort of patients further, stratified by age and according to whether they were treated with cemented or uncemented hemiarthroplasty. Although the numbers were relatively small (see Table 1), this analysis showed that elderly patients (> 70 years old) had a more favorable survivorship at 1 year when cemented monoblocks were compared to uncemented monoblocks (Figure 3) (p = 0.005 (patients 71–80 years old) and p < 0.001 (patients > 80 years old)). In the older age group (> 80), there was a higher 1-day mortality rate when cement was used (p < 0.001), but the significance of this difference was not apparent by 1 week (p = 0.5) or by 1 month (p = 0.9). The situation was even reversed by 1 year (p < 0.001), when cemented implants had a more favorable mortality rate.

### Cemented vs. uncemented modular components

**Figure 4.**
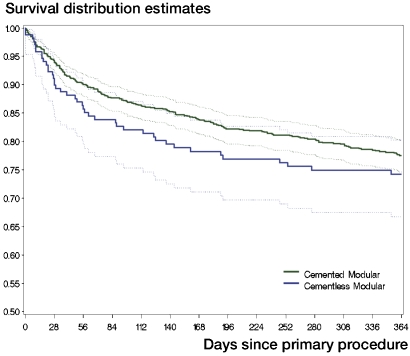
All-cause mortality in patients with cemented and uncemented modular components.

2,442 patients were treated with uncemented modular components, while 9,301 received cemented implants. There was no statistically significant difference in mortality at any time between the methods of fixation of modular implants (Table 5 and Figure 4).

**Table 5.** Hazard ratios (HRs) for risk of death, according to kind of fixation, for modular hemiarthroplasty

	HR (95% CI) (cementless vs. cemented)	p-value	No. of deaths	
			Cemented	Cementless
			(n = 9,301)	(n = 2,442)
1 day	0.48 (0.23–1.01)	0.05	63	8
1 week	1.18 (0.70–7.97)	0.5	207	44
1 month	0.91 (0.55–1.49)	0.7	501	119
1 year	0.89 (0.78–1.02)	0.09	1,665	381

## Discussion

Compared to uncemented procedures, we found reduced mortality at 1 year following a cemented procedure. At the outset of this study, it was our hypothesis that there would be similar mortality rates for cemented and uncemented hemiarthroplasty using a large, nationwide joint registry database. The fact that the data revealed a lower overall mortality rate at later times with cemented monoblock procedures was surprising. Together with the fact that implant survival from the AOA NJRR is increased for cemented implants than for uncemented implants into the medium term (AOA 2009) and the existence of previous work demonstrating improved functional outcome and pain scores with cemented implants (Khan et al. 2002a,b, Parker and Gurusamy 2004), it is becoming increasingly difficult to justify the continued preference of some surgeons for uncemented implants.

The strength of this study lies in the large numbers that were available for analysis. Due to high data completion rates and stringent data validation protocols by the AOA NJRR, the data are robust and easily lend themselves to this type of analysis.

There are many possible explanations for our findings. Firstly, a weakness of this study is that selection of implant fixation was not randomized. In that regard, other patient factors may have influenced the surgeon's decision to avoid cement, which may not have been adequately adjusted for in our analysis. For example, it has been shown that pre-existing cardiac disease is an independent risk factor for cement-related mortality (Parvizi et al. 2004). Other risk factors for increased perioperative mortality with hip fracture include age, sex, and comorbidities (Hannan et al. 2001, Jiang et al. 2005, Vestergaard et al. 2007b, Varez-Nebreda et al. 2008). The Australian Orthopaedic Association National Joint Replacement Registry does not collect comorbidity data, and in that regard we could not rule out the possibility that selection bias for fixation method influenced overall patient mortality. Our subcategorization of procedures into modular and monoblock components was an effort to control for this variable with a surrogate measure, as monoblock components are typically used in the frail elderly for quicker surgery and as there is less functional demand on the component postoperatively.

In a separate analysis, we found that there was a favorable survival rate at 1 year in patients aged 71–80 and > 80 when cemented monoblock implants were used rather than uncemented monoblock components. The reasons for this are unclear, but they may relate to selection of fixation method based on patient variables not captured by the AOA NJRR. For example, it is possible that elderly patients receiving cemented monoblock components are generally in better health than those treated with uncemented monoblock hemiarthroplasty, and are felt to be less likely to succumb to cement-related drop in systolic blood pressure intraoperatively. Alternatively, the opposite may be true—as for hip prostheses, generally fitter (younger, healthier) individuals receive cementless prostheses. There is also considerable state-to-state variability in preference for fixation method (AOA 2009), and individual hospital trends, which probably reflect different training and philosophies across the country. Further subanalysis of the relationship of these variables makes broad conclusions difficult, as patient numbers decrease with further subcategorization.

The cause of death was also not investigated in this study; thus, we could not directly link mortality to surgery-related issues. Certainly, 1-day and 1-week mortality are likely to be associated with perioperative factors. Consistent with our study, Foster et al. 2005) found a higher 30-day mortality rate in uncemented (9%) vs. cemented (1%) hemiarthroplasties in a retrospective review of 244 patients, despite similar ASA grades in both groups.

Parker and Gurusamy (2004) published a meta-analysis on the outcome of cemented hip arthroplasties vs. uncemented components for hip fracture, and found that mobility and pain at 1 year postoperatively was better in the cemented group. There was no difference in perioperative mortality in their analysis. This report included over 1,900 patients, although still substantially smaller than our study. The same findings were corroborated in a separate meta-analysis of 18 publications comparing cemented and uncemented arthroplasty for hip fractures (Khan et al. 2002b). Khan's group further compared 121 uncemented to 123 cemented Austin-Moore hemiarthroplasty patients done in 2 hospitals (Khan et al. 2002a). Patients treated with uncemented Austin-Moore implants had more pain, worse function in terms of walking and dependence on walking aids, and reduced capacity to perform activities of daily living compared to patients with cement fixation. There was no statistically significant difference in mortality or non-fatal medical complication rates related to type of fixation used. There were more intraoperative fractures (3/121 uncemented vs. 0/123 cemented), more dislocations (3/121 vs. 0/123), and a higher failure rate (numbers not reported) in patients with uncemented implants. In a single-institution audit, Singh and Deshmukh (2006) reported a higher overall reoperation and revision rate using uncemented Austin-Moore implants than when using cemented Thompson hemiarthroplasties. Patients treated with cemented implants also had a higher overall satisfaction rate relative to those with the uncemented stem. In a small, randomized study comparing cemented Thompson implants to uncemented Austin-Moore implants (Emery et al. 1991), pain and dependence on walking aids was less if the femoral component was fixed with cement. There was no difference in mortality or perioperative complications in either group. In a retrospective review of 107 patients treated with Thompson hemiarthroplasty for displaced femoral neck fractures, Sikorski and Millar (1977) failed to demonstrate any increased rate of mortality, myocardial infarction, cerebrovascular incident, cardiac failure, or postoperative hypotension whether or not cement was used. Similar findings have been reported with other comparisons of cemented and uncemented implants (Lausten and Vedel 1982, Lo et al. 1994).

Although pulmonary fat embolization is much less common with uncemented components, embolic events do occur (Orsini et al. 1987, Ries et al. 1993, Pitto et al. 1999), and this is probably related to increased intramedullary pressures during instrumentation of the femoral canal (Kallos et al. 1974, Orsini et al. 1987). Wenda et al. (1995) showed that reaming of the intramedullary canal produces pressures averaging 835 mmHg, and that only 200 mmHg is required for fat intravasation and embolization. This compares with maximum pressures of approximately 846 mmHg, demonstrated with introduction of cement into the femoral canal in a dog model by Orsini et al. 1987). In fact, there have been a few case reports outlining perioperative fat embolism syndrome and mortality due to fat embolization with uncemented hip arthroplasty (Arroyo et al. 1994, Gelinas et al. 2000). It is also known that intraoperative complications are higher with uncemented hemiarthroplasty, including iatrogenic femoral fracture (Foster et al. 2005, Weinrauch et al. 2006). A randomized, controlled trial investigating the prevalence of fat and bone marrow emboli in the lung based on right-atrium blood sampling showed similar prevalences with cemented and uncemented components (Kim et al. 2002). Furthermore, it has been shown that proper femoral canal lavage and vacuum suction reduce embolic events with cement implantation (Christie et al. 1995, Pitto et al. 1998). Modern cement techniques may therefore account for the lower incidence of perioperative mortality with use of cement compared to earlier studies.

In conclusion, this study shows a small but statistically significantly increased risk of mortality at 1 day when cement is used for monoblock hemiarthroplasty procedures. By 1 week, there is no longer a mortality advantage to avoiding cement, and by 1 year, mortality is less when cement is used. This may be due to a higher overall revision rate with uncemented monoblock components. When modular components are compared, there is no difference in mortality at any time analyzed, although there is a higher implant revision rate when uncemented components are used. These data support the use of cemented hemiarthroplasty components in patients with hip fracture.

## References

[CIT0001] AOA Australian Orthopaedic Association National Joint Replacement Registry Annual Report 2009. http://www.dmac.adelaide.edu.au/aoanjrr/publications.jsp.

[CIT0002] Arroyo JS, Garvin KL, McGuire MH (1994). Fatal marrow embolization following a porous-coated bipolar hip endoprosthesis. J Arthroplasty.

[CIT0003] Braithwaite RS, Col NF, Wong JB (2003). Estimating hip fracture morbidity, mortality and costs. J Am Geriatr Soc.

[CIT0004] Christie J, Burnett R, Potts HR, Pell AC (1994). Echocardiography of transatrial embolism during cemented and uncemented hemiarthroplasty of the hip. J Bone Joint Surg (Br).

[CIT0005] Christie J, Robinson CM, Singer B, Ray DC (1995). Medullary lavage reduces embolic phenomena and cardiopulmonary changes during cemented hemiarthroplasty. J Bone Joint Surg (Br).

[CIT0006] Conroy JL, Whitehouse SL, Graves SE, Pratt NL, Ryan P, Crawford RW (2008). Risk factors for revision for early dislocation in total hip arthroplasty. J Arthroplasty.

[CIT0007] Cooper C, Campion G, Melton LJ (1992). Hip fractures in the elderly: a world-wide projection. Osteoporos Int.

[CIT0008] Dearborn JT, Harris WH (1998). Postoperative mortality after total hip arthroplasty. An analysis of deaths after two thousand seven hundred and thirty-six procedures. J Bone Joint Surg (Am).

[CIT0009] Elliott J, Beringer T, Kee F, Marsh D, Willis C, Stevenson M (2003). Predicting survival after treatment for fracture of the proximal femur and the effect of delays to surgery. J Clin Epidemiol.

[CIT0010] Elmaraghy AW, Humeniuk B, Anderson GI, Schemitsch EH, Richards RR (1998). The role of methylmethacrylate monomer in the formation and haemodynamic outcome of pulmonary fat emboli. J Bone Joint Surg (Br).

[CIT0011] Emery RJ, Broughton NS, Desai K, Bulstrode CJ, Thomas TL (1991). Bipolar hemiarthroplasty for subcapital fracture of the femoral neck. A prospective randomised trial of cemented Thompson and uncemented Moore stems. J Bone Joint Surg (Br).

[CIT0012] Engesæter LB, Strand T, Raugstad TS, Husebø S (1984). Langeland,N. Effects of a distal venting hole in the femur during total hip replacement. Arch Orthop Trauma Surg.

[CIT0013] Foster AP, Thompson NW, Wong J, Charlwood AP (2005). Periprosthetic femoral fractures–a comparison between cemented and uncemented hemiarthroplasties. Injury.

[CIT0014] Gelinas JJ, Cherry R, MacDonald SJ (2000). Fat embolism syndrome after cementless total hip arthroplasty. J Arthroplasty.

[CIT0015] Hannan EL, Magaziner J, Wang JJ, Eastwood EA, Silberzweig SB, Gilbert M, Morrison RS, McLaughlin MA, Orosz GM, Siu AL (2001). Mortality and locomotion 6 months after hospitalization for hip fracture: risk factors and risk-adjusted hospital outcomes. JAMA.

[CIT0016] Icks A, Haastert B, Wildner M, Becker C, Meyer G (2008). Trend of hip fracture incidence in Germany 1995-2004: a population-based study. Osteoporos Int.

[CIT0017] Jiang HX, Majumdar SR, Dick DA, Moreau M, Raso J, Otto DD, Johnston DW (2005). Development and initial validation of a risk score for predicting in-hospital and 1-year mortality in patients with hip fractures. J Bone Miner Res.

[CIT0018] Kallos T, Enis JE, Gollan F, Davis JH (1974). Intramedullary pressure and pulmonary embolism of femoral medullary contents in dogs during insertion of bone cement and a prosthesis. J Bone Joint Surg (Am).

[CIT0019] Keating JF, Grant A, Masson M, Scott NW, Forbes JF (2006). Randomized comparison of reduction and fixation, bipolar hemiarthroplasty, and total hip arthroplasty. Treatment of displaced intracapsular hip fractures in healthy older patients. J Bone Joint Surg (Am).

[CIT0020] Khan RJ, MacDowell A, Crossman P, Datta A, Jallali N, Arch BN, Keene GS (2002a). Cemented or uncemented hemiarthroplasty for displaced intracapsular femoral neck fractures. Int Orthop.

[CIT0021] Khan RJ, MacDowell A, Crossman P, Keene GS (2002b). Cemented or uncemented hemiarthroplasty for displaced intracapsular fractures of the hip–a systematic review. Injury.

[CIT0022] Kim YH, Oh SW, Kim JS (2002). Prevalence of fat embolism following bilateral simultaneous and unilateral total hip arthroplasty performed with or without cement : a prospective, randomized clinical study. J Bone Joint Surg (Am).

[CIT0023] Koessler MJ, Fabiani R, Hamer H, Pitto RP (2001). The clinical relevance of embolic events detected by transesophageal echocardiography during cemented total hip arthroplasty: a randomized clinical trial. Anesth Analg.

[CIT0024] Lausten GS, Vedel P (1982). Cementing v. not cementing the Monk endoprosthesis. Injury.

[CIT0025] Leighton RK, Schmidt AH, Collier P, Trask K (2007). Advances in the treatment of intracapsular hip fractures in the elderly. Injury (Suppl 3).

[CIT0026] Lo WH, Chen WM, Huang CK, Chen TH, Chiu FY, Chen CM (1994). Bateman bipolar hemiarthroplasty for displaced intracapsular femoral neck fractures. Uncemented versus cemented. Clin Orthop.

[CIT0027] Lonnroos E, Kautiainen H, Karppi P, Huusko T, Hartikainen S, Kiviranta I, Sulkava R (2006). Increased incidence of hip fractures. A population based-study in Finland. Bone.

[CIT0028] Orsini EC, Byrick RJ, Mullen JB, Kay JC, Waddell JP (1987). Cardiopulmonary function and pulmonary microemboli during arthroplasty using cemented or non-cemented components. The role of intramedullary pressure. J Bone Joint Surg (Am).

[CIT0029] Parker MJ, Gurusamy K (2004). Arthroplasties (with and without bone cement) for proximal femoral fractures in adults. Cochrane Database Syst Rev.

[CIT0030] Parvizi J, Holiday AD, Ereth MH, Lewallen DG (1999). The Frank Stinchfield Award. Sudden death during primary hip arthroplasty. Clin Orthop.

[CIT0031] Parvizi J, Johnson BG, Rowland C, Ereth MH, Lewallen DG (2001). Thirty-day mortality after elective total hip arthroplasty. J Bone Joint Surg (Am).

[CIT0032] Parvizi J, Ereth MH, Lewallen DG (2004). Thirty-day mortality following hip arthroplasty for acute fracture. J Bone Joint Surg (Am).

[CIT0033] Pitto RP, Koessler M, Draenert K (1998). The John Charnley Award. Prophylaxis of fat and bone marrow embolism in cemented total hip arthroplasty. Clin Orthop.

[CIT0034] Pitto RP, Koessler M, Kuehle JW (1999). Comparison of fixation of the femoral component without cement and fixation with use of a bone-vacuum cementing technique for the prevention of fat embolism during total hip arthroplasty. A prospective, randomized clinical trial. J Bone Joint Surg (Am).

[CIT0035] Radcliff TA, Henderson WG, Stoner TJ, Khuri SF, Dohm M, Hutt E (2008). Patient risk factors, operative care, and outcomes among older community-dwelling male veterans with hip fracture. J Bone Joint Surg (Am).

[CIT0036] Ries MD, Lynch F, Rauscher LA, Richman J, Mick C, Gomez M (1993). Pulmonary function during and after total hip replacement. Findings in patients who have insertion of a femoral component with and without cement. J Bone Joint Surg (Am).

[CIT0037] Rinecker H (1980). New clinico-pathophysiological studies on the bone cement implantation syndrome. Arch Orthop Trauma Surg.

[CIT0038] Sikorski JM, Millar AJ (1977). Systemic disturbance from Thompson's arthroplasty: a age-matched and sex-matched controlled retrospective survey. J Bone Joint Surg (Br).

[CIT0039] Singh GK, Deshmukh RG (2006). Uncemented Austin-Moore and cemented Thompson unipolar hemiarthroplasty for displaced fracture neck of femur–comparison of complications and patient satisfaction. Injury.

[CIT0040] Varez-Nebreda ML, Jimenez AB, Rodriguez P, Serra JA (2008). Epidemiology of hip fracture in the elderly in Spain. Bone.

[CIT0041] Vestergaard P, Rejnmark L, Mosekilde L (2007a). Has mortality after a hip fracture increased?. J Am Geriatr Soc.

[CIT0042] Vestergaard P, Rejnmark L, Mosekilde L (2007b). Increased mortality in patients with a hip fracture-effect of pre-morbid conditions and post-fracture complications. Osteoporos Int.

[CIT0043] Weinrauch PC, Moore WR, Shooter DR, Wilkinson MP, Bonrath EM, Dedy NJ, McMeniman TJ, Jabur MK, Whitehouse SL, Crawford RW (2006). Early prosthetic complications after unipolar hemiarthroplasty. ANZ J Surg.

[CIT0044] Wenda K, Runkel M, Rudig L, Degreif J (1995). The effect of bone marrow embolization on the choice of procedure in the stabilization of femoral fractures. Orthopade.

